# Reduced Graphene Oxide-Extracellular Matrix Scaffolds as a Multifunctional and Highly Biocompatible Nanocomposite for Wound Healing: Insights into Characterization and Electroconductive Potential

**DOI:** 10.3390/nano12162857

**Published:** 2022-08-19

**Authors:** Javier Cifuentes, Carolina Muñoz-Camargo, Juan C. Cruz

**Affiliations:** Department of Biomedical Engineering, School of Engineering, Universidad de Los Andes, Carrera 1 No. 18A-12, Bogotá 111711, Colombia

**Keywords:** extracellular matrix, graphene oxide, reduced graphene oxide, electrostimulation therapy, wound healing, conductive scaffolds, tissue engineering, regenerative medicine

## Abstract

The development of novel regenerative technologies based on the implementation of natural extracellular matrix (ECM), or individual components of ECM combined with multifunctional nanomaterials such as graphene oxide and reduced graphene oxide, has demonstrated remarkable results in wound healing and tissue engineering. However, the synthesis of these nanocomposites involves great challenges related to maintaining the biocompatibility with a simultaneous improvement in their functionalities. Based on that, in this research we developed novel nanoengineered ECM-scaffolds formed by mixing small intestinal submucosa (SIS) with graphene oxide (GO)/reduced graphene oxide (rGO) to improve electrical conductivity while maintaining remarkable biocompatibility. For this, decellularized SIS was combined with GO to form the scaffold precursor for subsequent lyophilization, chemically crosslinking and in situ reduction. The obtained GO and rGO were characterized via Raman spectroscopy, Fourier transform infrared spectroscopy (FTIR), thermogravimetric analysis (TGA), X-ray diffraction (XRD), electrical conductivity testing and atomic force microscopy (AFM). The results confirm the suitable synthesis of GO, the effective reduction to rGO and the significant increase in the electrical conductivity (more than four orders of magnitude higher than bare GO). In addition, the graphene oxide/reduced graphene oxide-SIS scaffolds were characterized via Raman spectroscopy, FTIR, TGA, SEM, porosity assay (higher than 97.5% in all cases) and protein secondary structural analysis. Moreover, the biocompatibility of scaffolds was studied by standardized assays of hemolysis activity (less than 0.5%), platelet activation and deposition, and cell viability in Vero, HaCat and HFF-1 cells (higher than 90% for all evaluated cell lines on the different scaffolds). The obtained results confirm the remarkable biocompatibility, as supported by high hemocompatibility, low cytotoxicity and no negative impact on platelet activation and deposition. Finally, structural characteristics such as pore size and interconnectivity as well as superior cell attachment abilities also corroborated the potential of the developed nanoengineered ECM-scaffolds as a multifunctional nanoplatform for application in regenerative medicine and tissue engineering.

## 1. Introduction

The electrophysiological characteristics of cells is widely used to modify cellular activity [1,2]. The great knowledge around the influence of electrical fields in cellular behavior has allowed for the development of a great variety of therapies based on the application of external currents to stimulate cells, thereby generating different types of responses. For example, several studies have reported that cell growth, cell migration, wound healing, tissue regeneration, cell differentiation and membrane permeabilization were affected by the presence of exogenous electrical fields or the application of electrical stimulation that simulate physiological currents [1,2,3]. Due to the relation between the application of electrical fields and wound healing and tissue regeneration, an increasing number of studies have shown the development of innovative technologies based on combining both traditional acellular naturally derived and biosynthetic polymeric scaffolds with electrostimulation therapy [2,4,5]. This has attracted significant interest in the application of these combined technologies on the treatment of chronic wounds.

Chronic wounds are a specific type of wound that fail to progress through the normal reparative healing process and instead remain unhealed for more than 12 weeks [6]. These wounds are generally comorbidities of some increasingly prevalent pathologies such as diabetes mellitus, venous deficiencies, arterial perfusion, unrelieved pressure and obesity [7,8]. Currently, chronic wounds are a strongly relevant health problem with an increasing incidence that negatively impact the quality life of millions of people worldwide [7]. In the USA alone, this pathology represents an annual burden to the healthcare system of about USD 30 billion, which is mainly due to long periods of health care management and continuous application of palliative traditional therapies that are insufficient for solving the problem [7,8]. As a result, it is crucial to develop alternative therapies that go beyond palliative care and provide a long-lasting solution.

The emerging scaffolds/electrostimulation technologies are shown as very promising alternatives for the treatment of this kind of pathology [9]. This is because these technologies combine two different therapies that separately have shown important improvements in the treatment of difficult-to-manage wounds [3,4,6,10]. The first component of the therapy relies on scaffolds based on natural and biosynthetic extracellular matrices (ECMs) or individual components of ECMs, which have demonstrated significant improvement in tissue regeneration and healing of chronic wounds [10,11,12]. These special types of scaffolds are widely used due to their capability to generate a natural base for the cell migration, growth and proliferation [1,10]. The second component is electrostimulation, which requires the use of different biocompatible materials capable of conducting electrical currents. Some of the materials that fulfill these requisites include carbon-based materials [9], metals and metal oxide nanoparticles [2], and conductive polymers [13]. Electrostimulation has shown a powerful influence in the healing process by controlling the innate cellular electrophysiological characteristics [1,3,14].

Among the different materials used to develop ECM natural scaffolds, the porcine small intestinal submucosa (SIS) and porcine urinary bladder matrix have shown promising results, specifically in the treatment of venous ulcers and diabetic foot ulcers that are some of the most common wounds of difficult management [10]. In addition, one of the most relevant materials used to equip ECM scaffolds with the capacity to conduct electrical currents is the two-dimensional carbon nanomaterial graphene. Graphene is a two-dimensional monolayer of sp2 hybrid-bonded carbon atoms perfectly organized in a honeycomb structure [9]. This material demonstrates excellent electrical and thermal conductivities, high mechanical strength and remarkable optical properties [15,16].

This work is therefore dedicated to the development and characterization of innovative porcine small intestinal submucosa (SIS) and reduced graphene (rGO)/graphene oxide (GO) scaffolds as potential nanoplatforms for chronic wound healing and tissue engineering.

## 2. Materials and Methods

### 2.1. Materials

Sodium hypochlorite (10%), hydrogen peroxide (30%), sulfuric acid (50%), phosphoric acid (25%), hydrochloric acid (25%), ultra-pure ethanol (96%) and glacial acetic acid (99.5%) were purchased from PanReac AppliChem (Barcelona, Spain). Graphite flakes (99%), pepsin, N-[3-(dimethylamino)-propyl]-N’-ethylcarbodiimide hydrochloride (EDC) (98%), N-hydroxysuccinimide (NHS) (98%), ascorbic acid (99%), sodium chloride (99%), triton X-100, potassium permanganate (99%) and formaldehyde (36%) were purchased from Sigma-Aldrich (St. Louis, MO, USA). Dulbecco’s modified eagle’s medium (DMEM) and fetal bovine serum (FBS) were obtained from Biowest (Nuaillé, France). Penicillin/streptomycin (P/S) was purchased from Lonza (Basel, Switzerland). Hoechst 33342, Alexa Fluor 594 Phalloidin, LDH and alamar blue assay kits were purchased from Thermo Fisher Scientific (Waltham, MA, USA). Vero (ATCC^®^ CCL-81), HaCat (ATCC^®^ CCL-2404) and HFF-1 (ATCC^®^ SCRC-1041) cells were obtained from ATCC (St. Cloud, MN, USA).

### 2.2. SIS Obtention and Decellularization

SIS was obtained by mechanical removal of the tunica serosa, muscularis externa and tunica mucosa layers from a porcine small intestine and subsequently decellularized following the protocol reported by Sánchez-Palencia et al. [17]. Briefly, a decellularization solution consisting of sodium hypochlorite and hydrogen peroxide was used followed by PBS 1X and autoclaved type II water washes under constant stirring. The decellularized SIS was dried at room temperature in a laminar flow hood; next, the dry SIS was pulverized with a freeze-miller (6875 Freezer/Mill^®^, SPEX SamplePrep, Metuchen, NJ, USA) to obtain the SIS scaffold precursor powder.

### 2.3. Graphene Oxide Synthesis

Graphene oxide was synthetized following the protocol previously described by Marcano et al. [18]. Briefly, an acid solution consisting of sulfuric acid (90 mL) and phosphoric acid (10 mL) (9:1 ratio) was slowly added to 0.75 g of graphite flakes and 4.5 g of potassium permanganate. Subsequently, the resulting solution was heated at 50 °C under constant stirring for 12 h. Next, 150 mL of type I water ice cubes and 3 mL of hydrogen peroxide (30%) were added to the solution. Then, the solution was sonicated for 5 min (frequency 40 kHz, amplitude 38%), filtered by using a polyester fiber and centrifuged at 4000 rpm for 4 h. The supernatant was discarded, and the precipitated solid was resuspended in a wash solution made of 50 mL of type I water, 50 mL of extra pure ethanol and 50 mL of hydrochloric acid (30%). The resulting solution was sonicated, filtered and centrifuged using the conditions described above. This step was carried out three times. After this, GO was resuspended in a second wash solution made of 75 mL of type I water and 75 mL of extra pure ethanol, sonicated, filtered and centrifuged. Finally, GO was washed 2 times with type I water, sonicated, filtered and centrifuged. GO was finally lyophilized and stored at 4 °C.

### 2.4. SIS-rGO Scaffolds Fabrication

SIS powder was dissolved at 0.8% (*w*/*v*) in glacial acetic acid (0.5 M), pepsin (0.1% *w*/*v*) and GO (0.5 mg/mL) type I water solution. The resulting solution was left at room temperature and under constant agitation (500 rpm) for 48 h. SIS-GO scaffold precursor was lyophilized at −45 °C and 0.140 mbar for 36 h to obtain completely dried scaffolds. Next, the scaffolds were crosslinked by immersion in a 25 mM EDC and 25 mM NHS ultra-pure ethanol solution for 24 h. The crosslinked scaffolds were washed 5 times with type I water and, finally, lyophilized under the same conditions. To reduce the GO, SIS-GO crosslinked scaffolds were immersed in 2% (*w*/*v*) ascorbic acid type I water solution and left at 50 °C under constant agitation for 12 h [19]. Finally, SIS-rGO scaffolds were washed 10 times with type I water and then they were lyophilized under the same conditions. Scaffolds were sterilized via ethylene oxide and stored at 4 °C until further use.

### 2.5. GO, rGO and Scaffolds Characterization

GO and rGO were characterized by Raman spectroscopy using a XPlora Raman Horiba confocal (Horiba Scientific, Kyoto, Japan). Fourier transform infrared spectroscopy (FTIR), thermogravimetric analysis (TGA) and X-ray diffraction (XRD) were used to determine the correct synthesis, oxidation level, and the effectiveness of the reduction process. AFM microscopic analysis was performed to analyze the GO structure using an MFP-3D-BIO AFM (Asylum Research, Santa Barbara, CA, USA). Moreover, conductivity of GO and rGO was determined by the four-point probe method using a S-302-4 Four Point Resistivity Probing Equipment (Signatone, Gilroy, CA, USA). SIS, crosslinked SIS, SIS-GO and SIS-rGO scaffolds were characterized via Fourier transform infrared spectroscopy (FTIR) and thermogravimetric analysis (TGA) (TA Instruments, New Castle, DE, USA). Infrared spectra were recorded using an A250 FT-IR (Bruker, Germany) (4000–400 cm^−1^ range and 2 cm^−1^ spectral resolution). TGA analysis was performed by using a temperature ramp of 10 °C/min from 25 to 400 °C in a nitrogen atmosphere (gas flow: 100 mL/min). XRD analysis was performed using a Malvern-Panalytical-Empyrean model (45 kV, 40 mA) (Malvern Panalytical, Malvern, UK) at a range of 5–80°. Scaffolds morphologies were analyzed by microscopy image analysis of scanning electron microscope (SEM) micrographs Tescan Lyra3 (TESCAN, Brno, Czech Republic).

### 2.6. Scaffolds Porosity

Porosity (π) was estimated by the liquid displacement method [20]. Briefly, each scaffold was submerged in 3 mL (V1) of type I water and left for 5 min to allow water absorption. Then, the new volume (V2) (V1+ volume of scaffold) was recorded. Finally, liquid-impregnant scaffold was removed, and the remaining volume is V3. Porosity is calculated according to π = (V1 − V3)/(V2 − V3).

### 2.7. Cell Viability in Vero, HaCat and HFF-1 Cells

Cell viability was evaluated via Alamar blue assay in Vero (ATCC^®^ CCL-81, kidney cells from African green monkey-*Cercopithecus aethiops*) HaCat (ATCC^®^ CCL-2404, human skin keratinocytes-*Homo sapiens*) and HFF-1 cells (ATCC^®^ SCRC-1041, human skin fibroblast-*Homo sapiens*). For this, completely dry-sterile scaffolds (SIS, SIS-GO and SIS-rGO) were put in a 24-well microplate and 200,000 cells suspended in 100 µL of supplemented DMEM media were seeded on the top of each scaffold. Cell-Scaffolds were incubated at 37 °C, 5% CO_2_, for 3 h to allow cell adhesion and then 300 µL of supplemented DMEM media was added to each well. Scaffolds were then incubated at 37 °C, 5% CO_2_ for 48 h. After the incubation time, scaffolds were removed out of the culture microplate. Next, scaffolds were transferred a 24 microplate with 270 µL of supplemented DMEM media and 30 µL of the Alamar Blue dye and allowed to incubate for 3 h. The resulting 300 µL solution was removed from each sample and then, the fluorescence was measured at room temperature in a Horiba spectrofluorometer FuoroMax 4 (Horiba Scientific, Japan) using an excitation and emission wavelength of 560 and 590 nm, respectively. A calibration curve was performed to determine cell number by correlating a known cell number with the fluorescent intensity of the solution.

### 2.8. Hemolysis Assay

Blood sample was obtained from a healthy human donator. Erythrocytes were collected by centrifugation at 1800 rpm for 5 min, plasma was discarded and then erythrocytes were washed 5 times with NaCl solution (0.9% (*w*/*v*)) and 1 time with PBS (1X). A total of 1 mL of washed erythrocytes was suspended in 9 mL of PBS (1X) to prepare the stock dispersion (cellular density of 700,000 erythrocytes/μL). Hemolysis induced by crosslinked SIS scaffolds, SIS-GO and SIS-rGO scaffolds (5 mm diameter × 2 mm thickness) was by direct contact and by contact with extracts of the materials. PBS (1X) was used as negative control and triton X-100 (10% (*v*/*v*)) as positive control. Briefly, to evaluate direct contact hemolytic effect, scaffolds were suspended in 100 μL of PBS (1X) and then 100 μL of erythrocytes solution was added and incubated at 37 °C, 5% CO_2_, for 1 h. Samples were centrifuged at 1800 rpm for 5 min. 100 μL of each supernatant was placed in a 96-well microplate and read at 450 nm in a microplate reader. On the other hand, extract hemolytic effect was tested by suspending the scaffolds in 200 μL of PBS (1X) for 21 days and then 100 μL of the supernatants was placed in a 96-well microplate with 100 μL of erythrocytes solution and incubated at 37 °C, 5% CO_2_, for 1 h. Finally, samples were centrifuged and 100 μL of each supernatant was read following the conditions described previously.

### 2.9. Platelet Activation and Adhesion Assay

Platelets were obtained by centrifuging fresh human blood at 1000 rpm for 15 min (blood sample was collected in sodium citrate vacutainer tubes to avoid aggregation). Crosslinked SIS scaffolds, SIS-GO scaffolds and SIS-rGO scaffolds were tested. Scaffolds (5 mm diameter × 2 mm thickness) were placed in a 96-well plate and then 100 μL of platelet-rich plasma was added to each well. Samples were incubated at 37 °C, 5% CO_2_, for 30 min; then, scaffolds were washed 3 times with PBS 1X. Next, platelets were fixed with 4% (*v*/*v*) formaldehyde in PBS at 20 °C for 15 min. Samples were washed 3 more times with PBS (1X) and then dehydrated in graded ethanol solutions (from 35% to 100%). Finally, the samples were dried at room temperature and sputter-coated with gold. Platelet adhesion was observed using an SEM Tescan Lyra3 (TESCAN, Brno, Czech Republic). Furthermore, LDH assay was carried out to quantify the platelet activation. Briefly, scaffolds were seeded with platelet-rich plasma as described previously; after 30 min of incubation, 10 μL of triton X-100 (1% (*v*/*v*)) were added. Samples were centrifuged at 1800 rpm for 5 min and then 50 μL of the supernatant was extracted and placed in a 96-well plate with 50 μL of LDH reagent. Platelet-rich plasma with triton X-100 (1% (*v*/*v*)) and platelet-rich plasma with PBS (1X) were used as positive control and negative control, respectively. Finally, samples were read in a microplate reader at 595 nm.

### 2.10. Cell Distribution and Morphology Analysis

Cell distribution and morphology into the scaffolds were analyzed by following the protocol reported by Girão et al. [9]. Briefly, scaffolds with cylindrical dimensions 10 mm (diameter) × 2 mm (thickness) were tested. First, the different samples were washed with sterile culture media (DMEM) supplemented with 10% (*v*/*v*) of fetal bovine serum (FBS) and 1% (*v*/*v*) of penicillin streptomycin for 15 min. The scaffolds were placed in a 24-well plate (one scaffold in one well) and seeded with 100.000 Vero cells suspended in 100 µL culture media. For this, cells were added to the top of the scaffolds and next, incubated for 3 h at 37 °C and 5% CO_2_ to allow cell adhesion. After this, 100 µL of supplemented culture media were added and, finally, incubated for 24 h under the same conditions. Cell distribution and morphology were studied by fluorescence analysis. Briefly, cells were fixed with formaldehyde 4% (*v*/*v*) for 10 min. Then, cell membranes were permeabilized using triton X-100 (1% (*v*/*v*)) for 5 min. Scaffolds were washed with PBS (1X) to remove the excess of Triton X-100 and formaldehyde. Cell nuclei were stained with Hoechst 33342 and actin filaments with Phalloidin Alexa Fluor 594, incubated for 60 min at 4 °C, and finally washed with PBS (1X). Finally, the scaffolds were immediately observed in an Olympus FV1000 confocal microscope (Olympus, Tokyo, Japan), with 20× (0.75 NA, UPlanSApo) and 60× (1.42 NA, oil PlanApo N). Images were taken with 350 nm and 540 nm excitation wavelength and 470 nm and 565 nm emission wavelength. Image analysis was performed in Fiji.

### 2.11. Statistical Analysis

All the obtained results are presented as mean ± standard deviation. Statistical analysis was performing by using the software Graph Pad Prism V 6.01 software (GraphPad Software, San Diego, CA, USA). Statistical comparisons were carried out employing ANOVA followed by the Tukey’s Multiple Comparison test. Results with a *p*-value ≤ 0.05 (*) were considered statistically different. Symbol * corresponds to statistically significant difference with a *p*-value in the range of 0.01 ≤ *p*-value ≤ 0.05, ** to statistically significant difference with a *p*-value in the range of 0.001 ≤ *p*-value < 0.01, *** to *p*-value in the range of 0.0001 ≤ *p*-value ≤ 0.001 and **** to *p*-value < 0.0001.

## 3. Results and Discussion

### 3.1. GO and rGO Characterization

**Figure 1:**
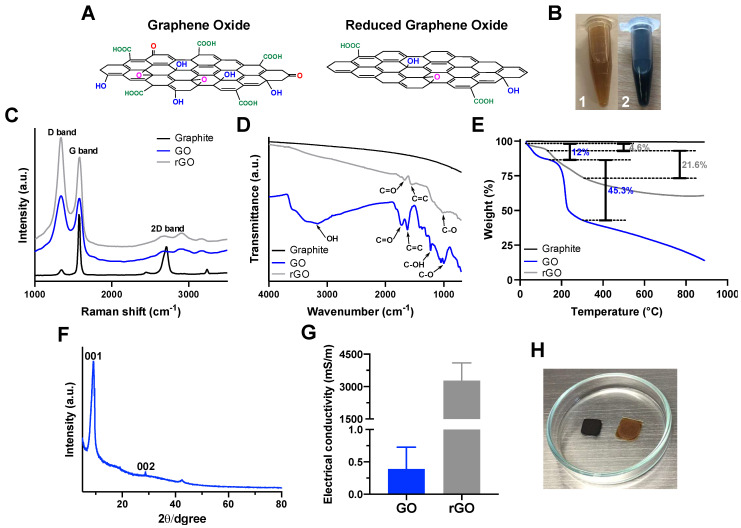
Characterization of GO and rGO. (**A**) Schematic representation of the chemical structure of GO and rGO. (**B**) GO/ultra−pure ethanol solution (1) and rGO/ultra−pure ethanol solution (2). (**C**) Raman spectra (514 nm) of graphite, GO and rGO. (**D**) FTIR spectra of graphite, GO and rGO. (**E**) TGA thermograms for graphite, GO and rGO. (**F**) XRD pattern of GO. (**G**) Electrical conductivity of GO and rGO films. (**H**) GO and rGO films (deposited on glass slides).

The chemical structures of GO and rGO are shown in Figure 1A, where it is possible to observe the predominant carboxyl, epoxy and hydroxyl functional of the as-synthesized GO. After reduction, a decrease in such groups is expected for rGO. Figure 1B shows the resulting suspensions of GO and rGO in ethanol. GO suspension is dark brown to light yellow depending on the concentration. After reduction, the color of the suspension turns black (rGO suspension). These results agree well with the reported optical properties of GO and rGO [21,22,23]. This optical change allows a qualitative verification of a structural shift due to the reduction of the GO.

Correct synthesis, high oxidation level and reduction of GO were confirmed by Raman, FTIR, TGA and XRD analysis as shown in Figure 1C–F, respectively. Raman analysis was performed to contrast the structure of GO after and before the reduction. Furthermore, the Raman spectrum of graphite was also measured in order to determine structural changes after the oxidation process (correct synthesis of GO). Figure 1C shows the Raman spectra of graphite, GO and rGO. The spectrum of graphite shows a strong G band at 1570.8 cm^−1^ due to the first-order scattering of E_2g_ mode [21,22,23]. Graphite spectrum also presents a small D band at 1341.7 cm^−1^ that is related to the presence of defects in the material and a 2D band at 2705.1 cm^−1^ that is widely used to evaluate the c-axis orientation and the stacking order of the graphite along the same axis [21]. GO spectrum shows two strong signals, G and D bands, at 1588.9 and 1345.8 cm^−1^, which can be assigned to the sp^2^ and sp^3^ hybridizations of carbon, respectively [24]. The G peak shifted to 1588.9 cm^−1^, most likely due to the oxygenation of graphite [22], and a more intense D band appeared in response to the reduction in size of in-plane sp^2^ domains (graphite). This can be attributed to the presence of defects and distortions of the sp^2^ domains generated by the complete oxidation of the material [21,22]. The intensity of the 2D band decreases as the oxidation level increases, which provides further evidence for the notion that the intensity shift in the 2D band of GO compared to graphite is due to the presence of GO with a high oxidation level [21]. After the reduction of GO, the G band is shifted to a lower wave number (1580.6 cm^−1^), which agrees well with previous research studies [24,25]. This change was attributed to the recovery of the hexagonal network of carbon atoms with defects [22,26]. Structural changes after reduction and oxidation were also studied by looking at the D/G intensity ratio. The I(D)/I(G) ratio for GO corresponds to 1.04, which is most likely due to a high oxidation level [21]. After reduction, the I(D)/I(G) ratio for rGO approached a value of 1.2. This greater ratio generally indicates an increase in the number of small sp^2^ domains [27] and can be a consequence of the increase in the D band intensity, which, in turn, can be attributed to the large number of defects that are commonly present on the rGO surface [25]. In addition, the increase in the I(D)/I(G) ratio after reduction can be also due to an increase in the degree of disorder in carbon-based materials, which is typically observed in the reduction process [25]. Our findings agree well with previous reports on the synthesis of GO with a high oxidation level. Moreover, the significant changes in the GO structure confirmed that the reduction of GO to obtain rGO proceeded correctly.

Figure 1D shows the FTIR spectra of the GO, rGO and graphite. The GO spectrum shows multiple peaks associated to oxygen-derived species [25]. This confirms the presence of different functional groups such as carboxyl, epoxy, hydroxyl, and alkoxy on the surface of GO. This, compared to the graphite precursor spectrum, confirms the correct oxidation reaction [24]. The peaks at 1721 and 3400 cm^−1^ correspond to stretching vibrations of the C=O and OH groups [24]. The peaks at 1226 and 1050 cm^−1^ can be assigned to the stretching vibration of C-OH and the bending vibration of C-O, respectively [24]. The peak at 1620 cm^−1^ correspond to C=C aromatic stretching vibration [25]. Due to the reduction process, the rGO spectrum presents an important decrease in the number of peaks associated with oxygen-derived species (Figure 1D). However, the presence of C=O and C-O stretching vibrations in the rGO spectrum confirms the partial reduction of GO.

The thermal stability of GO, rGO and precursor graphite were evaluated by using thermogravimetric analysis as shown in Figure 1E. The TGA thermogram of graphite shows a high thermal stability up to 900 °C. The GO thermogram shows a first mass loss (12%) at 100 °C, which is most likely due to the presence of bound water. This is followed by an important weight loss (45.3%) at about 200 °C, which might be related to the removal of oxygen-rich species [22,27,28]. This data provide further evidence of the high level of oxidation of GO. In contrast, the first mass loss of rGO (100 °C) approached 4.6%, which most likely corresponds to the removal of water. The decrease in weight loss for water compared with the material prior to reduction is due to the high hydrophobicity of rGO [28]. After chemical reduction with ascorbic acid, the obtained rGO shows a second weight loss of (21.6%) at 200 °C and might be due to the remaining oxygen-rich species. This weight loss is higher than the previously reported for rGO [28], which can be explained by the partial reduction of GO. Additionally, XRD pattern of GO present the characteristic peaks at 9.3° attributed to the (001) plane, and at 29.1° it corresponded to the (002) plane (Figure 1F).

In order to study the electrical conductivity of GO before and after the reduction, the four-point probe method was used. Figure 1G shows the electrical conductivity (mS/m) of GO and rGO films (Figure 1H). GO presents a very low electrical conductivity with an average of 0.4 mS/m; this agrees well with the previously reported values of conductivity [29]. The low electrical conductivity of GO is due to its high oxidation level. As reported elsewhere, the presence of multiple oxygen functional groups on the surface leads to an important decrease in the conductivity [30]. After the reduction, electrical conductivity of rGO was 3300 mS/m, which is more than 4 orders of magnitude higher than that of GO (Figure 1G). This confirms that as the oxygen functional groups of GO decreased in content, the electrical conductivity properties are significantly improved. However, the electrical conductivity of the obtained rGO is lower than previously reported values, which can be attributed to the incomplete reduction process [29,30].

**Figure 2:**
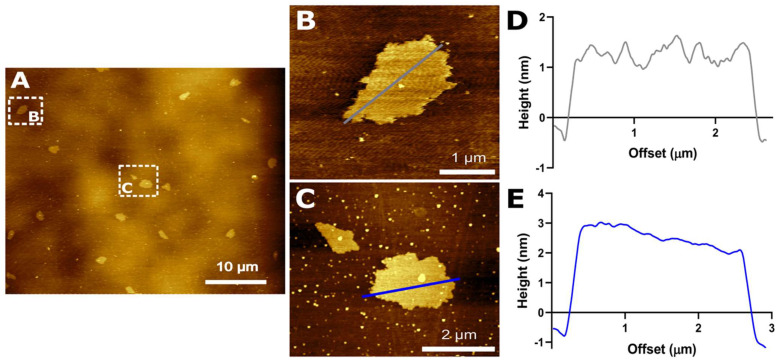
(**A**) AFM image of GO. (**B**,**C**) correspond to more detailed observations of GO nanosheets in Figure 2A. The height profiles of (**B**,**C**) GO nanosheets are shown in (**D**,**E**), respectively.

AFM analysis was carried out to characterize the topography of GO sheets deposited on a SiO_2_ substrate. Figure 2A shows the AFM image of an aqueous solution of GO (Type I water, 0.5 mg/mL). The height profile of two different sheets is presented in Figure 2D,E. The results show the typical sheet-like morphology and it is possible to identify different levels of stacking. In this regard, Figure 2B shows a low level while Figure 2C shows an increased level. The average height of the obtained GO sheets corresponds to 1 nm, which closely agrees with the previously reported thickness of GO sheets [21,27]. The stacking of GO sheets is the result of aggregation or self-assembly during lyophilization [21].

### 3.2. Scaffolds Characterization

To understand the interactions between GO and SIS and to establish the impact of the in situ GO reduction on the scaffold properties, Raman, TGA and FTIR analysis were carried out.

**Figure 3:**
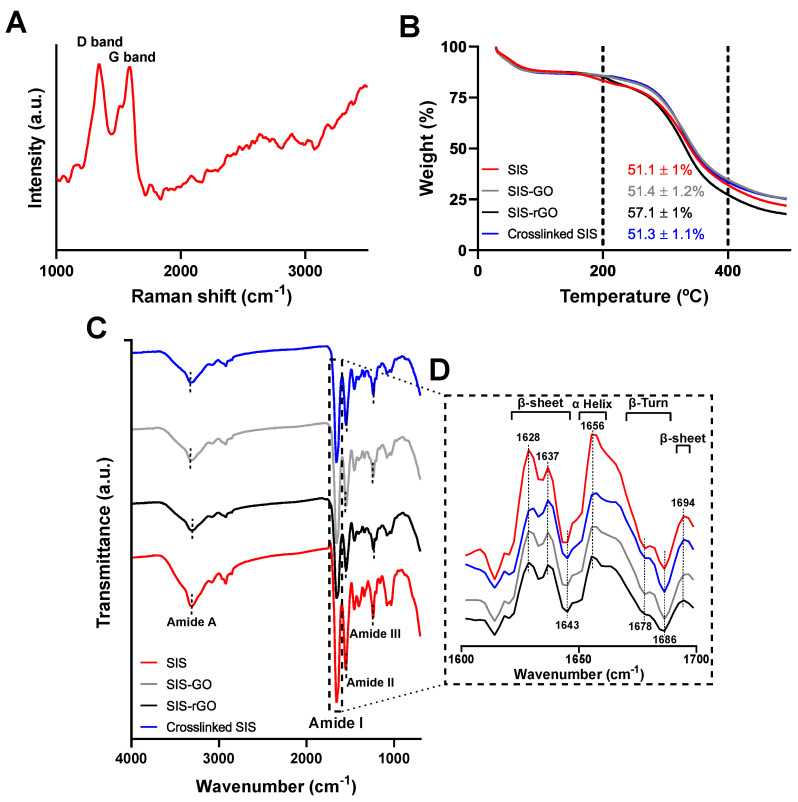
Characterization of the scaffolds. (**A**) Raman spectra of SIS-rGO scaffold. (**B**) TGA and (**C**) FTIR analysis of the developed nanocomposites. (**D**) Second derivative of the FTIR spectra in range of the amide I band (1700−1600 cm^−^^1^).

Figure 3A shows the Raman spectrum of SIS-rGO scaffolds, where two strong bands, D (1348.6 cm^−1^) and G (1586.1 cm^−1^), are clearly visibly. The I(D)/I(G) ratio corresponds to 1.01, which agrees well with reported values for the in situ reduction of GO using ascorbic acid on collagen scaffolds [19]. This result indicates the successful assembly of GO and the correct in situ reduction. TGA analysis was used to evaluate the thermal stability of SIS, crosslinked SIS, SIS-GO and SIS-rGO scaffolds (Figure 3B). Major thermal degradation of collagen, the main component of SIS, occurs in the range of 300–400 °C [31,32,33]. Because of this, the effect of crosslinking, GO conjugation and in situ reduction on the thermal stability of collagen was then evaluated by analyzing TGA thermograms in the same temperature range. First weight loss of all the samples occurs at 200 °C. The mean weight loss in temperature range of interest, corresponded to 51.1% for SIS, 51.3% for crosslinked SIS, 51.4% for SIS-GO, and 57.1% for SIS-rGO. Statistical analysis suggests that there is no significant difference between the obtained results for all the scaffolds. Moreover, thermograms for all the scaffolds were similar, thereby indicating that crosslinking, GO conjugation and in situ reduction had no significant impact on the thermal stability of collagen compared to pristine SIS scaffolds. These results agree well with the results obtained by Kang et al. [31] and Lee et al. [33].

Figure 3C,D show the FTIR and the second derivative analysis of SIS, crosslinked SIS, SIS-GO and SIS-rGO scaffolds, respectively. FTIR spectra for all the scaffolds present a peak at 3330 cm^−1^ that is attributed to N=H stretching vibration (Amide A). A peak was also observed at 1655 cm^−1^, which is due to the C=O stretching vibration (Amide I), the one at 1550 cm^−1^ to C–O and N−H combined bending vibration (Amide II) and that at 1250 cm^−1^ to C–N stretching vibration. Finally, N−H bending and C–C stretching (Amide III) vibrations of collagen type I (main component of the scaffolds) were also identified [33]. It is important to note that the peak at 1640 cm^−1^ is not only related to the amide I band, but also to the C=C and C=O (carbonyl) stretching vibrations of GO and rGO [9]. In addition, the peak situated at 1240 cm^−1^ can be associated with the stretching vibration of the epoxy group present in both GO and rGO [9]. All the FTIR spectra are very similar due to the overlapping of the different peaks present on the SIS spectrum (related to collagen type I), GO and rGO spectra. Second derivative analysis of the amide I band of the FTIR spectra were conducted to determine possible changes in the secondary structure of collagen type I protein (Figure 3D) [34]. No significant changes were detected, which indicated that secondary structural changes in collagen were negligible after crosslinking, GO conjugation and the final reduction step.

**Figure 4:**
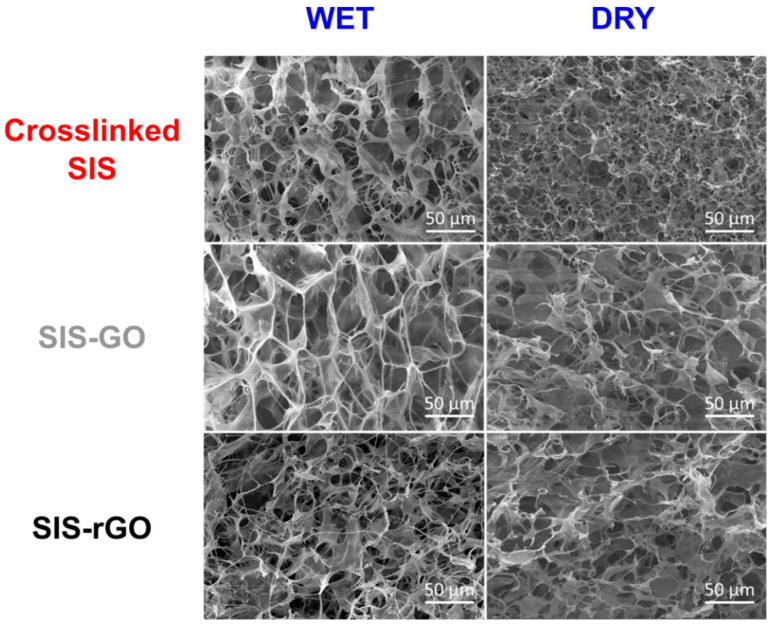
SEM images of hydrated and completely dry scaffolds. Pores appear homogenously distributed and well interconnected.

Microscopic features of the developed nanocomposites were studied via SEM. Figure 4 shows SEM images of the different scaffolds before and after hydration. Pore size in all the scaffolds is larger in the wet state than in the dried state. However, the pore size of crosslinked SIS and SIS-GO scaffolds after the hydration (38 ± 8 µm and 45 ± 11 µm, respectively) almost doubled with respect to the dried state (18 µm ± 5 µm and 19 µm ± 7 µm, respectively). This was not the case for rGO, where pore size after the hydration remained almost unchanged (dry 18 ± 4 µm, wet 23 ± 7 µm). The different scaffolds exhibit similar pore sizes in the dry state; however, after hydration differences are quite significant. This important discrepancy could be due to the different interactions between the scaffolds and the water. Crosslinked SIS scaffolds have an important increase in pore size as a result of its high hydrophilicity. This was also the case for the SIS-GO scaffolds, which are rich in free-oxygen functional groups that might improve the interaction with the water, thereby allowing them to absorb more water and consequently to have a significant increase in pore size [32]. SIS-rGO scaffolds are, however, highly hydrophobic, mainly due to the absence of polar groups on the surface of rGO [26]. This feature considerably limits the interactions with the water, thereby preventing water absorption and consequently leading to smaller changes in pore size. Additionally, images revealed that pores within the scaffolds are well distributed and highly interconnected, which is an essential characteristic for cell adhesion, migration and proliferation [32].

**Figure 5:**
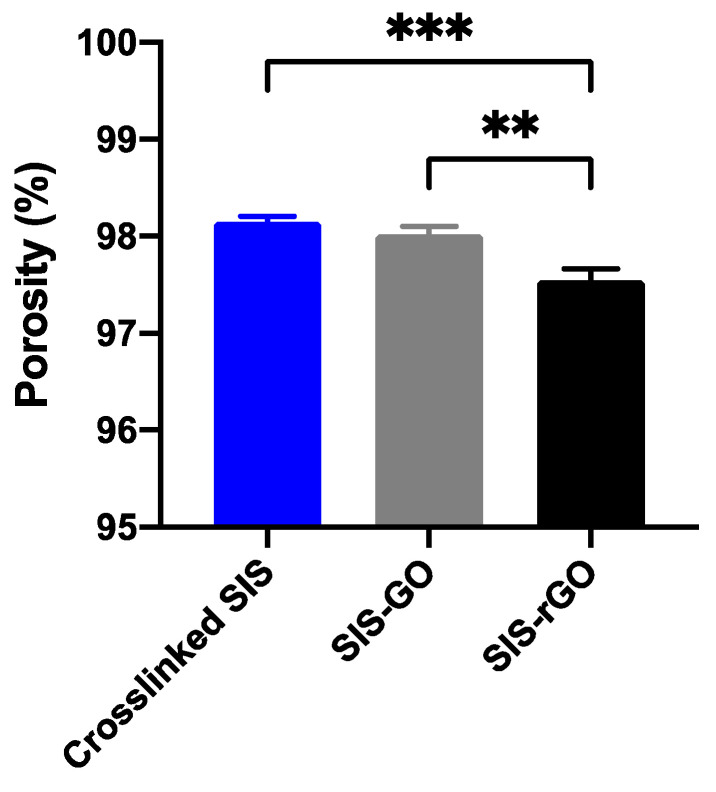
Porosity of the scaffolds calculated by the liquid displacement method. Results with a *p*-value ≤ 0.05 (*) were considered statistically different. Symbol * corresponds to statistically significant difference with a *p*-value in the range of 0.01 ≤ *p*-value ≤ 0.05, ** to statistically significant difference with a *p*-value in the range of 0.001 ≤ *p*-value < 0.01, *** to *p*-value in the range of 0.0001 ≤ *p*-value ≤ 0.001 and **** to *p*-value < 0.0001.

Porosity of the scaffolds was studied by the liquid displacement method. Figure 5 shows that the porosities of the scaffolds are higher than 97%. However, there is a statistically significant difference between the SIS-rGO and the crosslinked SIS and SIS-GO scaffolds. This result suggests that reductions in ascorbic acid and temperature are likely to impact the porosity. Nevertheless, all the scaffolds exhibit a porosity that is sufficient for proper cell distribution and migration as well as penetration of body fluids, which is beneficial for increasing the amount of nutrients available to the cells [20].

### 3.3. Scaffolds Biological Characterization

**Figure 6:**
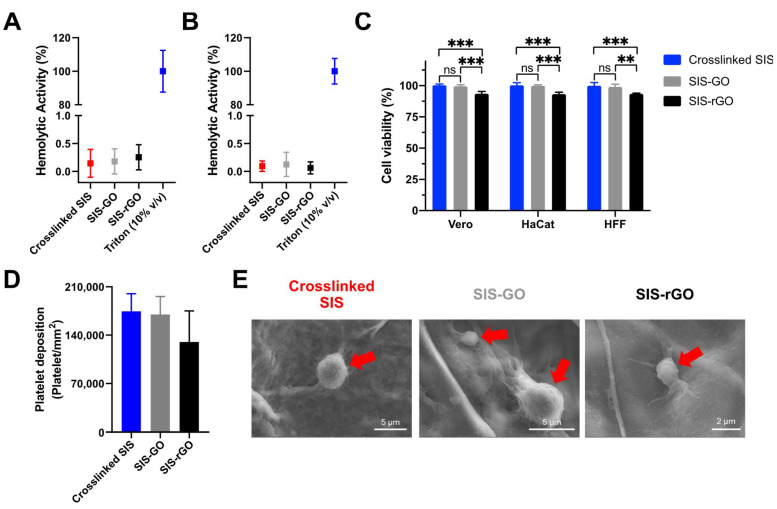
Hemolytic effect of the nanocomposites tested by extracts (**A**) and direct contact (**B**). (**C**) Cell viability of the different scaffolds in Vero, HaCat and HFF-1 cells (Alamar blue assay). Platelet activation tested by LDH quantification assay (**D**) and SEM images of platelet deposition (**E**) evaluated in crosslinked SIS, SIS-GO and SIS-rGO scaffolds. Red arrows show activated platelets. Results with a *p*-value ≤ 0.05 (*) were considered statistically different. Symbol * corresponds to statistically significant difference with a *p*-value in the range of 0.01 ≤ *p*-value ≤ 0.05, ** to statistically significant difference with a *p*-value in the range of 0.001 ≤ *p*-value < 0.01, *** to *p*-value in the range of 0.0001 ≤ *p*-value ≤ 0.001 and **** to *p*-value < 0.0001.

To obtain a first insight into the potential of the developed scaffolds for tissue regeneration, cell proliferation and chronic wound healing applications, we carried out hemolysis, cytocompatibility in Vero cells, and platelet interaction tests. Figure 6A,B show the hemolytic activity of the different scaffolds as tested in both extracts of the materials and by direct contact, respectively. All the results demonstrated an average hemolytic below 1% for both measurements. According to the ISO standard 10993: 2009, the obtained results confirmed the high hemocompatibility of the SIS scaffolds, as previously discussed elsewhere [32,35]. This was also the case after conjugation of GO [32,36]. Figure 6C shows the cytocompatibility of the nanocomposites in Vero, HaCat and HFF-1 cells. All the scaffolds showed cell viability higher than 90%, and in the case of crosslinked SIS and SIS-GO, it even approached 100%. This is easily explainable by the superior biocompatibility of SIS, and the enhanced cell attachment by interactions with the oxygen functional groups and the cell membrane proteins [19]. The cell viability reduction for SIS-rGO can be attributed to the high hydrophobicity of rGO (due to the absence of oxygen-rich functional groups), which limits interactions with cell membrane proteins, and consequently the ability of cells to attach [19]. This suggests that cells preferentially attach to highly hydrophilic surfaces, a result that agrees well with the findings of Ramsay et al. [37]. Finally, Figure 6D,E show the interaction of the scaffolds with human platelets through platelet deposition and activation assays. Figure 6D indicates that there is no statistically significant difference between the platelet deposition for crosslinked SIS, SIS-GO and SIS-rGO scaffolds. This implies that both GO and rGO had no significant effect on the platelet deposition and aggregation over the well-known effect of collagen [38]. Figure 6E shows SEM images of platelet adhesion on the different scaffolds.

### 3.4. Cell Morphology and Distribution

**Figure 7:**
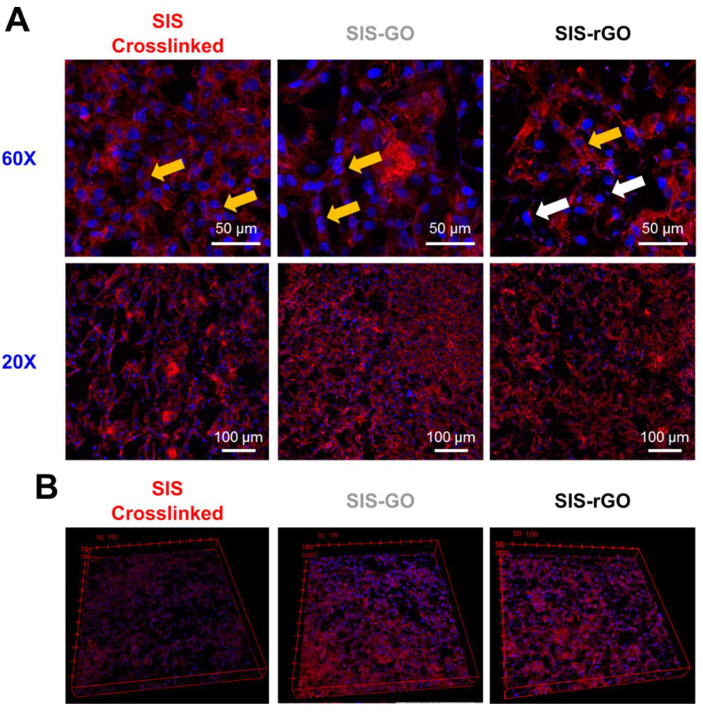
(**A**) Analysis of Vero cell morphology via confocal imaging. Yellow arrows present cells that exposed the typical elongated morphology confirming strong adhesion to the matrix, and white arrows expose cells with irregular round-shape morphology indicating poor adhesion to the matrix. (**B**) Three-dimensional reconstruction of cell distribution of Vero cells in all the developed scaffolds (confocal images 20×). Cell nuclei were stained with Hoechst 33342 (Blue) and actin filaments with Alexa Fluor 594 Phalloidin (Red).

Morphology of Vero cells was studied 24 h after seeding by confocal image analysis. As shown in Figure 7A, Vero cells maintain their polygonal shape in all the different scaffolds. Moreover, yellow arrows indicate the presence of cells strongly attached to the scaffold, thereby indicating that the developed scaffolds are likely to support living Vero cells and promote cell spreading and proliferation. However, SIS-rGO scaffolds present some cells with a spherical shape (white arrows), thereby suggesting no proper attachment in those areas, which can be attributed to the hydrophobicity imparted by the rGO. Nevertheless, it appears that the fewer oxygen groups on the surface of rGO allow for the successful interaction with Vero cells in some areas (Figure 7A, yellow arrows) [9]. Furthermore, cell nuclei are homogeneously distributed along the scaffolds in the xy plane, which suggests a higher cell concentration surrounding the pores. Finally, Figure 7B shows 3D reconstructions of the scaffolds. It is possible to observe a uniform distribution of the cells, however, due to the high optical density of the samples, observation in the *z* axis was impeded at a depth higher than 70 µm from the surface.

## 4. Conclusions

Multifunctional SIS-GO and SIS-rGO nanohybrid scaffolds were successfully developed in this study. The obtained results demonstrate the remarkable biocompatibility in terms of hemocompatibility, negligible cytotoxicity (Vero, HaCat and HFF-1 cells) and no negative impact on platelet activation and deposition. Additionally, structural characteristics such as pore size, distribution, and interconnectivity, as well as their notable cell attachment abilities, also corroborated the potential of the developed nanoengineered scaffolds for their suitable application in several fields, such as regenerative medicine, tissue engineering and wound healing. Future research should be focused on testing the potential of the scaffolds in specific electrostimulation applications and relevant physiological environments. Finally, it is very important to analyze the effect of the developed scaffolds on cell proliferation and cytotoxicity during long-term stimulation experiments, as well as in additional cell lines and eventually in vivo.

## Data Availability

Not applicable.
